# Supplementary material for: The WD40 domain of ATG16L1 is required for its non‐canonical role in lipidation of LC3 at single membranes
Source: EMBO J. 2018 Jan 9;37(4):e97840. doi: 10.15252/embj.201797840 (PMC5813257; doi:10.15252/embj.201797840)
Supplement: Supplementary file 1 — Expanded View Figures PDF [file EMBJ-37-e97840-s001.pdf]

Expanded View Figures

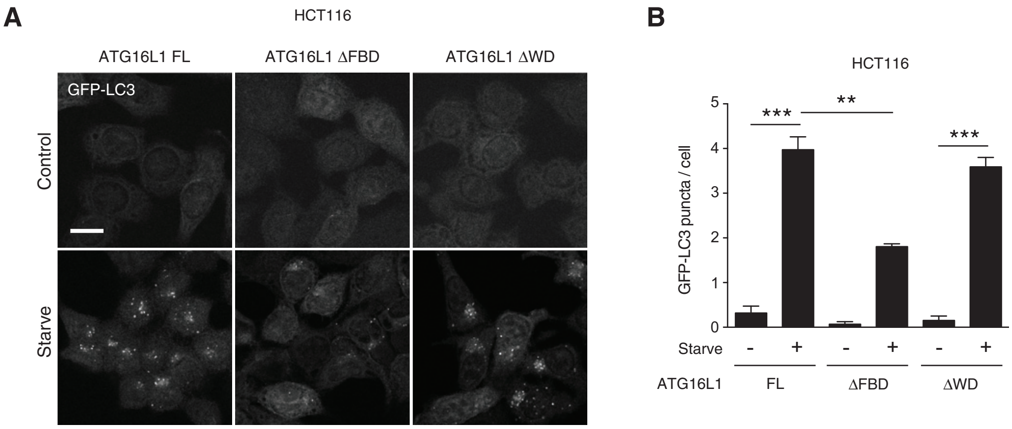

Figure EV1. The WD40 CTD of ATG16L1 is not required for canonical autophagy.

A Confocal images of GFP-LC3-expressing HCT116 *ATG16L1*$^{-/-}$ complemented with full-length (FL), ΔFBD or ΔWD ATG16L1 under fed or starvation conditions. Scale bar: 10 μm.

B Quantification of GFP-LC3 puncta in ATG16L1-complemented HCT116 cells under fed or starvation conditions.

Data information: In (B), data are presented as mean ± SEM from three independent experiments. ***$P < 0.0004$, **$P < 0.002$ (Student's *t*-test).

 © 2018 The Authors

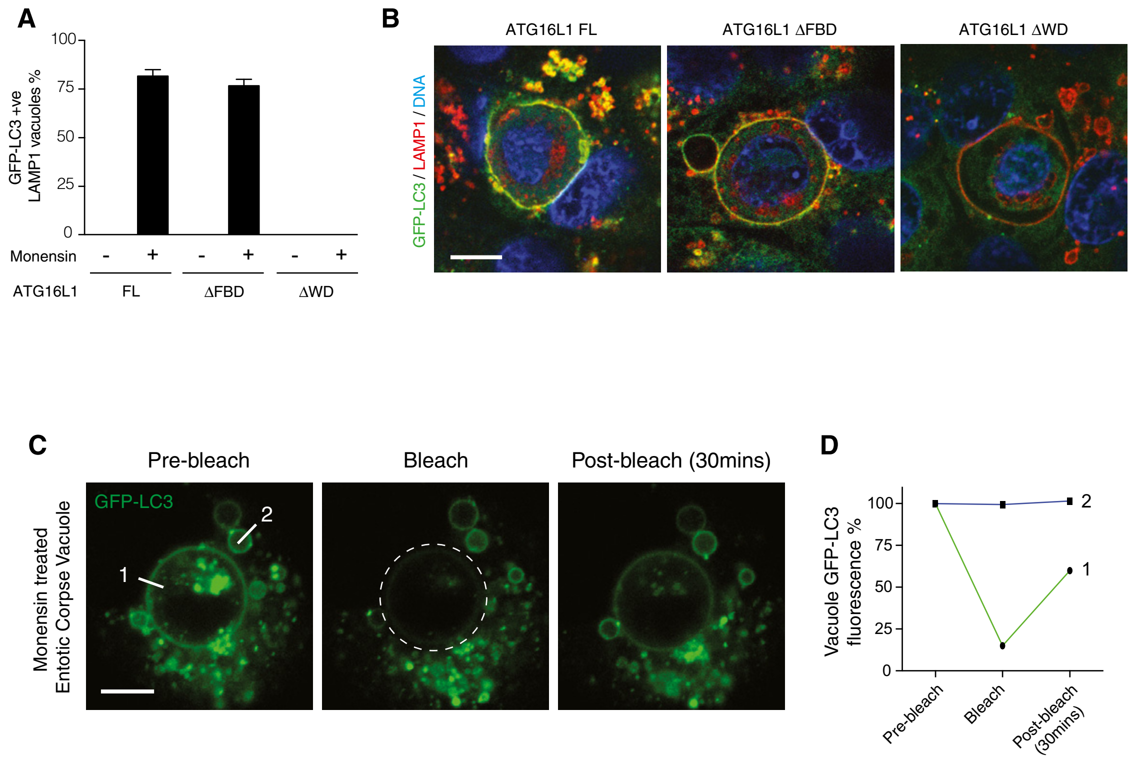

Figure EV2. Monensin-induced LC3 lipidation to entotic corpse vacuole requires the WD40 CTD of ATG16L1.

A Quantification of GFP-LC3 recruitment to LAMP1-positive entotic corpse-containing vacuoles in MCF10A *ATG16L1*$^{-/-}$ cells complemented full-length (FL), ΔFBD or ΔWD ATG16L1 ± monensin (100 μM, 1 h).

B Representative confocal images of entotic corpse-containing vacuoles in GFP-expressing MCF10A cells treated with 100 μM monensin for 1 h and stained for LAMP1 (red) and DNA (blue). Scale bar: 10 μm.

C Representative sequence images from FRAP analysis of GFP-LC3 on entotic corpse-containing vacuoles treated with monensin (100 μM, 1 h). The region marked by a broken-line circle was photobleached, and the recovery of fluorescence at line 1 and 2 was monitored. Scale bar: 10 μm.

D Quantification of GFP fluorescence at line 1 and 2 from (C).

Data information: In (A), data are presented as mean ± SEM from three independent experiments.

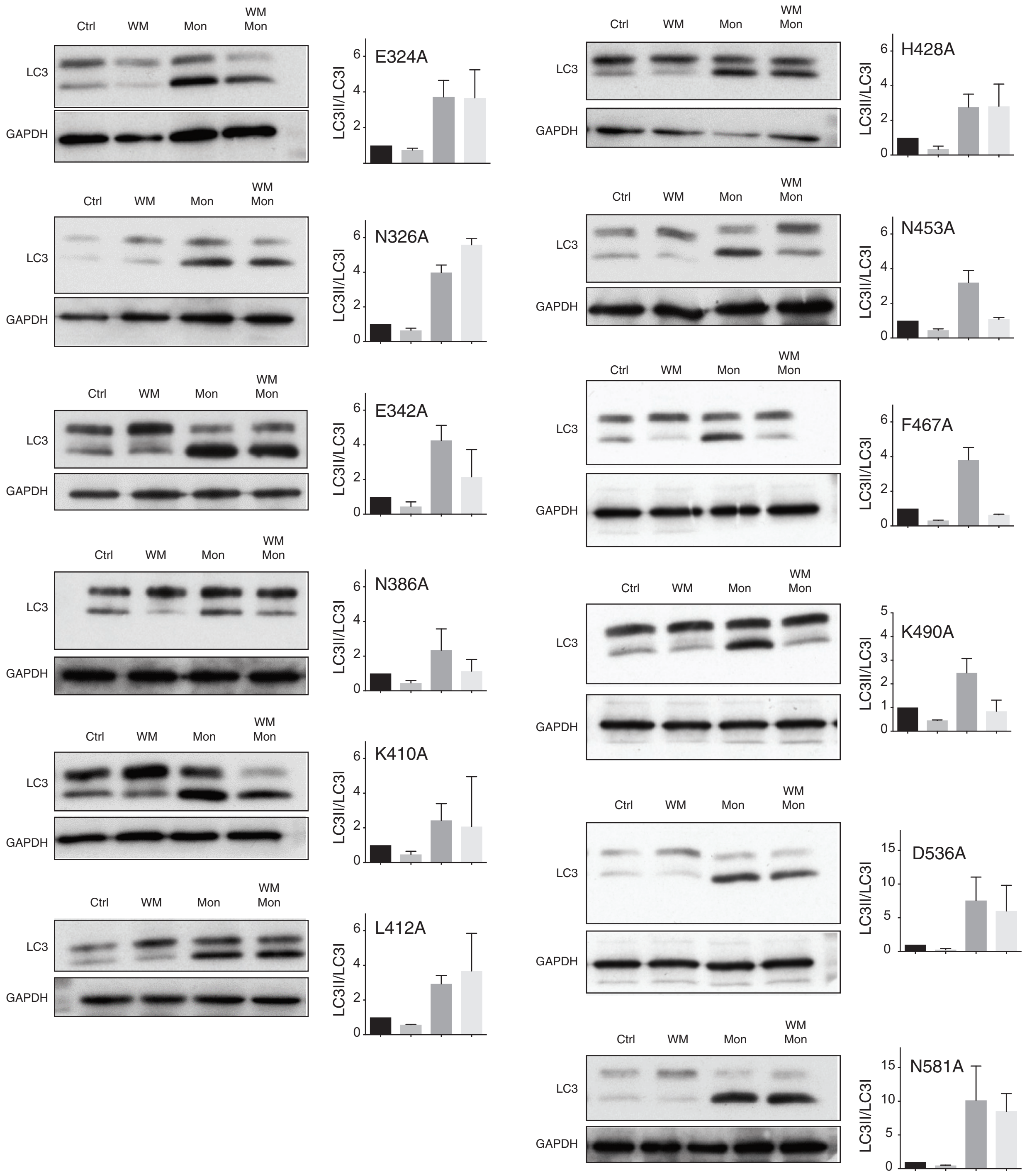

Figure EV3. Screening of ATG16L1 WD40 CTD mutants for monensin-induced non-canonical autophagy.

Western blotting of LC3 from ATG16L1-complemented HCT116 cells treated with wortmannin (WM, 67 μM), monensin (Mon, 100 μM) or both for 1 h. To the side is the quantification of LC3-II/LC3-I ratios. Data are presented as mean ± SEM from three independent experiments.

 © 2018 The Authors

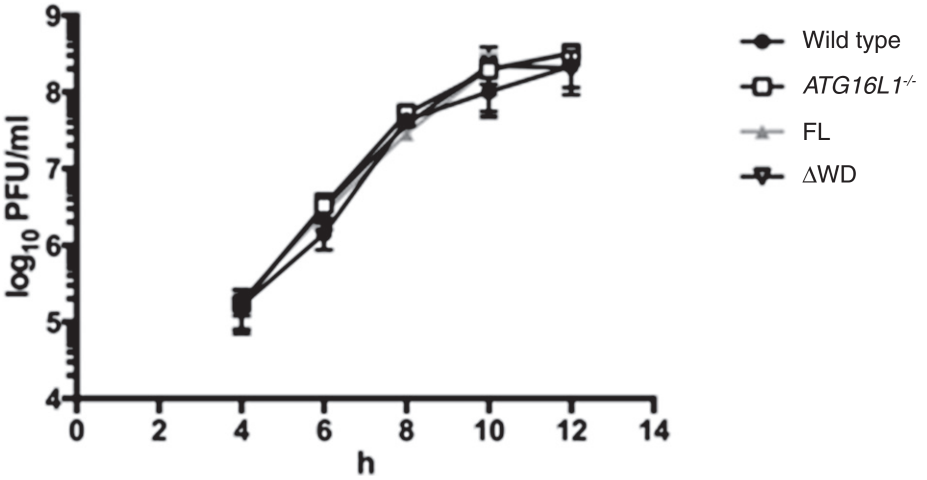

Figure EV4. The effect of ATG16L1 WD40 CTD on IAV titre.

Wild-type or *ATG16L1*$^{-/-}$ HCT116 cells complemented with full-length (FL) or ΔWD ATG16L1 were infected with IAV PR8 at an moi of 5 PFU per cell. At the indicated timepoints, the titre of virus in the supernatant was determined by plaque assay on MDCK cells. Error bars are standard deviation from 3 replicates.

© 2018 The Authors

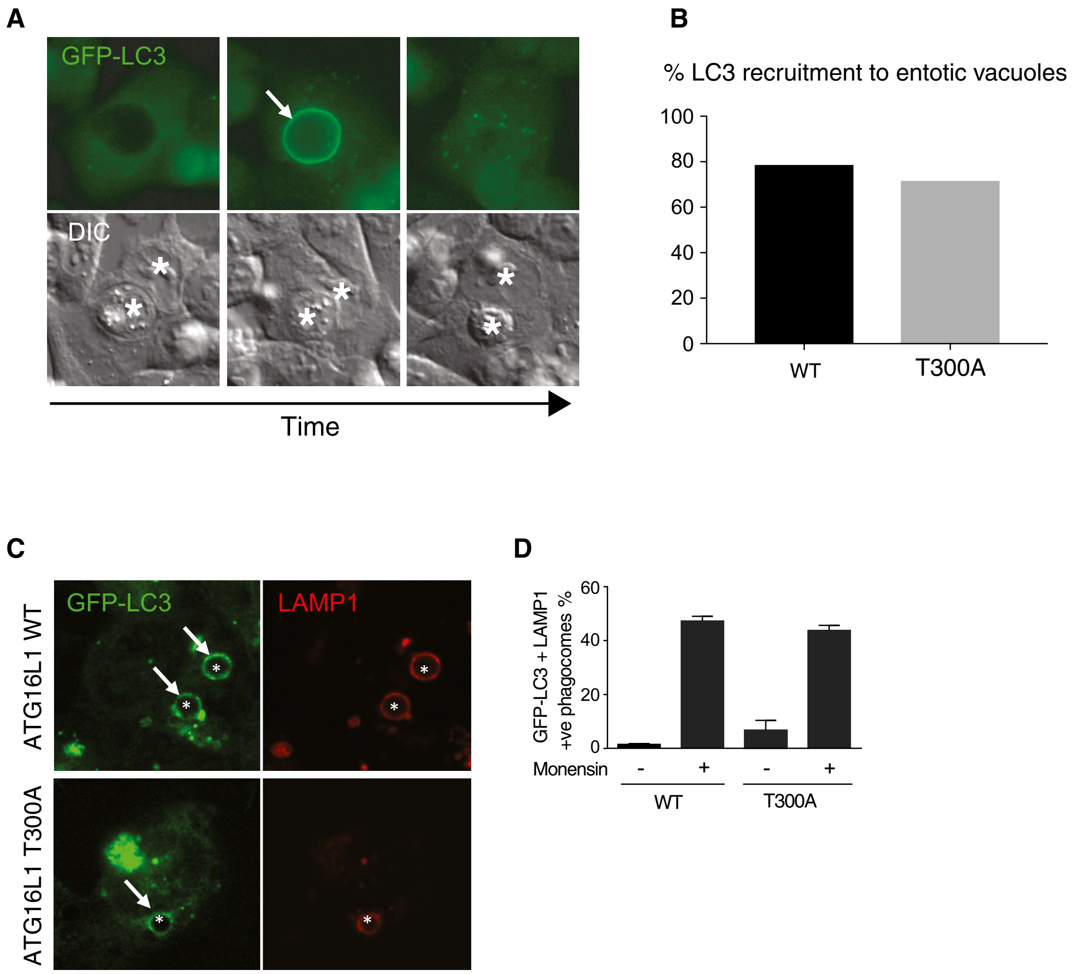

Figure EV5. ATG16L1 T300A mutation has no effect on non-canonical autophagy-associated LC3 lipidation.

A Wide-field time-lapse images showing the recruitment of GFP-LC3 to entotic vacuoles in wild-type HCT116 cell-in-cell structures. Arrow indicates GFP-LC3 recruitment to entotic vacuole. Asterisks indicate the host and internalised cells.

B Quantification of GFP-LC3 recruitment to entotic vacuoles over 20 h in wild-type and ATG16L1 T300A-expressing HCT116 cells. Data represent analysis of > 15 structures.

C Confocal images of latex bead-containing phagosomes in monensin-treated (100 μM, 1 h) GFP-LC3-expressing HCT116 cells expressing wild-type or T300A ATG16L1. Asterisks indicate latex bead-containing phagosomes, and arrows indicate GFP-LC3-positive phagosomes.

D Quantification of GFP-LC3 recruitment to LAMP-1-positive phagosomes in wild-type and T300A HCT116 cells. 100 phagosomes were counted per experiment.

Data information: In (D), data are presented as mean ± SEM from three independent experiments.

 © 2018 The Authors
